# Ghrelin Inhibits the Differentiation of T Helper 17 Cells through mTOR/STAT3 Signaling Pathway

**DOI:** 10.1371/journal.pone.0117081

**Published:** 2015-02-06

**Authors:** Yanhui Xu, Ziru Li, Yue Yin, He Lan, Jun Wang, Jing Zhao, Juan Feng, Yin Li, Weizhen Zhang

**Affiliations:** 1 Department of Physiology and Pathophysiology, Peking University Health Science Center, Key Laboratory of Molecular Cardiovascular Science, Ministry of Education, Beijing, 100191, China; 2 Department of Surgery, University of Michigan, Ann Arbor, MI, United States of America; Seoul National University College of Pharmacy, KOREA, REPUBLIC OF

## Abstract

Enhanced activity of interleukin 17 (IL-17) producing T helper 17 (Th17) cells plays an important role in autoimmune and inflammatory diseases. Significant loss of body weight and appetite is associated with chronic inflammation and immune activation, suggesting the cross talk between immune and neuroendocrine systems. Ghrelin has been shown to regulate the organism immune function. However, the effects of ghrelin on the differentiation of Th17 cells remain elusive. In the present study, we observed the enhanced differentiation of Th17 cells in spleens of growth hormone secretagogue receptor 1a (GHSR1a)^-/-^ mice. Treatment of ghrelin repressed Th17 cell differentiation in a time- and concentration-dependent manner. Phosphorylation of mammalian target of rapamycin (mTOR) and signal transducer and activator of transcription 3 (STAT3) was increased in the spleens of GHSR1a^-/-^ mice. Activation of mTOR signaling by injection of Cre-expressiong adenovirus into tuberous sclerosis complex 1 (TSC1) ^loxp/loxp^ mice increased the differentiation of Th17 cells in spleen, which was associated with an increment in the phosphorylation of STAT3. Activation of mTOR signaling by leucine or overexpression of p70 ribosome protein subunit 6 kinase 1 (S6K1) activated mTOR signaling in isolated T cells, while reversed the ghrelin-induced inhibition of iTh17 cell differentiation. In conclusion, mTOR mediates the inhibitory effect of ghrelin on the differentiation of Th17 cells by interacting with STAT3.

## Introduction

T helper 17 (Th17) cells, an important subset of T cells, exert pro-inflammatory function to enhance the development of various autoimmune and inflammatory diseases [1]. Th17 cells are characterized by the expression of intracellular RAR-related orphan receptor γt (RORγt) [2] and signal transducer and activator of transcription 3 (STAT3) [3], and the production of cytokines such as interleukin (IL)-17A, IL-17F, IL-21 and IL-22 [4]. The increase in Th17 cells are found in many autoimmune and inflammatory diseases such as multiple sclerosis, inflammatory bowel diseases, rheumatoid arthritis, atherosclerosis and immunometabolic diseases [5,6,7,8]. Reducing the amount of Th17 cells may be a promising therapeutic strategy for these diseases.

Ghrelin, a 28 amino acid peptide hormone, is mainly secreted from gastric X/A-like cells. It comes in two isoforms, acylated and des-acylated. Upon synthesis, ghrelin is acylated on its third serine residue and this octanoylation appears to be critical for its binding to the classical receptor, growth hormone secretagogue receptor 1a (GHSR1a) [9,10]. Besides the effects on food intake and promotion of growth hormone secretion, ghrelin mediates or influences a wide variety of physiological functions, including glucose homeostasis, pancreas function, cardiovascular function, memory, sleep [11,12]. Recent studies also demonstrate that ghrelin is critical in the regulation of immune function. Ghrelin improves experimental autoimmune encephalomyelitis (EAE) [13] and autoimmune colitis [14] by inhibiting the mRNA and protein expression of inflammatory cytokines such as IL-1, IL-6 and tumor necrosis factor (TNF)-α, enhancing the expression of anti-inflammatory cytokine IL-10, and inhibiting apoptosis of immune cells [15,16]. A recent study suggests that expression of pro-inflammation cytokines including IL-17 increases in ghrelin knocked down T cells [17]. However, whether and how ghrelin affects the differentiation of Th17 cells remains unknown.

The differentiation of Th17 cells is controlled by a number of intracellular signaling cascades and a complex network of transcription factors. The nuclear orphan receptor RORγt coordinates the diverse cytokine-induced signals to control Th17 cell differentiation [18]. STAT3 has emerged as an important regulator of the differentiation of Th17 cells [3]. Mammalian target of rapamycin (mTOR) in T cells integrates environmental clues including co-stimulatory molecule engagement (CD28), growth factors, amino acid and insulin to regulate the differentiation of T cells [19,20]. Rapamycin, the inhibitor of mTOR, has been reported to expand regulatory T cells and to inhibit the differentiation of Th17 cells [21].

In present study, we found that ghrelin inhibited Th17 cell differentiation both *in vivo* and *in vitro*. The effect of ghrelin might be attributed to the reduced activation of STAT3 and mTOR signaling pathway. These data verify the immune function of ghrelin and indicate a new mechanism of ghrelin to exert its anti-inflammatory effect.

## Materials and Methods

### Animals

GHSR1a-/- mice were obtained from Shanghai Animal Model Centre. Tuberous sclerosis complex 1 (TSC1)^loxp/loxp^ mice were from Jackson Laboratories. Six-week-old male C57BL/J6 mice were purchased from the Animal Center of Peking University Health Science Center (Beijing, China). This study was carried out in strict accordance with the recommendations in the Guide for the Care and Use of Laboratory Animals of the Health Science Center of Peking University. The protocol was approved by the Committee on the Ethics of Animal Experiments of Peking University Health Science Center.

### Cell Sorting and Induction of Th17 Differentiation *in vitro*

Total splenic T cells were purified with nylon fiber colon (Polyscience, America) following the manufacturer’s instructions. For FACS-sort of splenic CD4^+^ T cells, cell suspensions were stained with anti-CD4 FITC (Miltenyi Biotec, Bergisch Gladbach, Germany) and sorted on a FACStar Plus (Becton Dickinson) instrument. Cells were reanalyzed immediately after sort and purities were >95%. Then 2 ×10^6^ T cells were seeded in 24 well plates containing plate-bound anti-CD3 (1 μg/ml, BD Pharmagen, Franklin Lakes) and soluble anti-CD28 (1 μg/ml, BD Pharmagen, Franklin Lakes) antibodies, and cultured with RPMI 1640 medium (Hyclone, Carlsbad, CA) containing 10% inactivated fetal bovine serum (Hyclone, Carlsbad, CA) For Th17 cell differentiation, cultures were treated with transforming growth factor (TGF)-β1 (5 ng/ml, Pepro Tech), and IL-6 (20 ng/ml, Pepro Tech), together with anti-interferon-γ (IFN-γ) antibody (5 μg/ml, R&D Systems, Minneapolis, MN) and anti-IL-4 antibody (5 μg/ml, R&D Systems, Minneapolis, MN) as indicated [22,23]. The differentiation of Th17 cells were confirmed with flow cytometry assay, without affecting the percentage of Treg cells (S1 Fig.). Ghrelin (Phoenix), leucin (Sigma), Colivelin (Abgent) were used at the doses indicated. For flow cytometry, RT-PCR and Western Blot, cells were collected 4 days later.

### Adeno-virus Infection

Isolated splenic T cells were induced to differentiate into Th17 cells as described previously [22]. Fourty eight hours after differentiation, cells were infected with adenovirus expressing p70 ribosome protein subunit 6 kinase 1 (Ad-S6K1) or green fluorescent protein (Ad-GFP).

### Flow Cytometry Analysis

For IL-17A staining, Leukocyte Activation Cocktail and BD GolgiPlug (mixture of brefeldin A, PMA, ionomycin, BD Pharmagen, Franklin Lakes) were added. Six hours later, cells were collected and suspended in fixation/Permeabilization solution (BD Cytofix/Cytoperm kit-BD pharmingen), and intracellular IL-17A staining with Alexa Fluor 647-tagged anti-IL-17A antibody (BD Pharmagen, Franklin Lakes) was performed according to the manufacturer’s instructions.

### RNA extraction and Real-Time RT-PCR

Cells were collected 4 days after the induction of Th17 cell differentiation, and total RNA extracted with TRIzol reagent (Invitrogen, Carlsbad, CA) according to the manufacturer’s instructions. Then, AMV reverse transcription system (Promega, Madison, WI) was introduced to perform the reverse transcription of 1 μg of RNA per sample. Real-time PCR amplifications involves an Mx3000 multiplex quantitative PCR system (Stratagene Corp, La Jolla, CA) and SYBR Green I reagent. All amplification reactions carried out for 40 cycles were performed in duplicate (an initial stage of 7 min at 95°C, followed by a three-step cycle of 20 s at 94°C, 45 s at 60°C, and 40 s at 72°C). The accuracy of PCR products was confirmed by sequencing of the amplicons. The relative target mRNA levels normalized to that of the internal control β-actin were assessed with Stratagene Mx3000 software. Primers used in this study were shown in Table 1.

**Table 1:** List and sequences of primers used in RT-PCR experiments.

	Upstream primer (5’-3’)	Downstream primer (5’-3’)
Mouse RORγt	AATGGAAGTCGTCCTAGTCAG	CCGTGTAGAGGGCAATCTCA
Mouse IL-17A	CCTCAGACTACCTCAACCG	CTCCCTCTTCAGGACCAG
Mouse FoxP3	TCCTTCCCAGAGTTCTTCCAC	ACTTGTGCAGGCTCAGGTTGT
Mouse GATA3	CATTACCACCTATCCGCCCTATG	CACACACTCCCTGCCTTCTGT
Mouse Tbx21	CAACAACCCCTTTGCCAAAG	TCCCCCAAGCAGTTGACAGT
Mouse IFNγ	TTTTCAGCTCTGCATCGTTTTGGGT	CCTTGAAACAGCATCTGACTCCTT
Mouse β-actin	ATCTGGCACCACACCTTC	AGCCAGGTCCAGACGCA

### ELISA Analysis

Supernatant of cell culture or serum of mice was collected, ELISA was performed with mouse IL-17A quantifying kit (eBioscience, San Diego, CA) with an indicated sensitivity of below 4 pg/ml. The kit was used following the the manufacturers’ instructions.

### Western Blot Analysis

Immunoblotting was performed as described previously. Briefly, T-cell lysis samples containing the same amount of protein were resolved in 10% SDS-PAGE. The membranes were incubated with primary antibodies, then IRDye 700DX-conjugated secondary antibodies (Rockland Inc, Gilbertsville, PA). The immunofluorescence signal was detected by the Odyssey infrared imaging system (LICOR Biosciences, Lincoln, NB). The primary antibodies include rabbit-anti-total or phosphorylated STAT3 antibodies, rabbit-anti-total or phosphorylated S6 antibody (Cell Signal Technology, Danvers, MA) and rabbit-anti-β-actin (Santa Cruz, CA).

### Statistical Analysis

All data were expressed as mean±SEM or original data representing one of at least three independent experiments. One-way ANOVA followed by Newman-Keul’s post hoc test was used to compare multiple groups. Unpaired Student t-test was performed to compare two groups. P < 0.05 was considered statistically significant.

## Results

### Differentiation of Th17 cells was enhanced in GHSR1a^-/-^ mice

**Fig 1:**
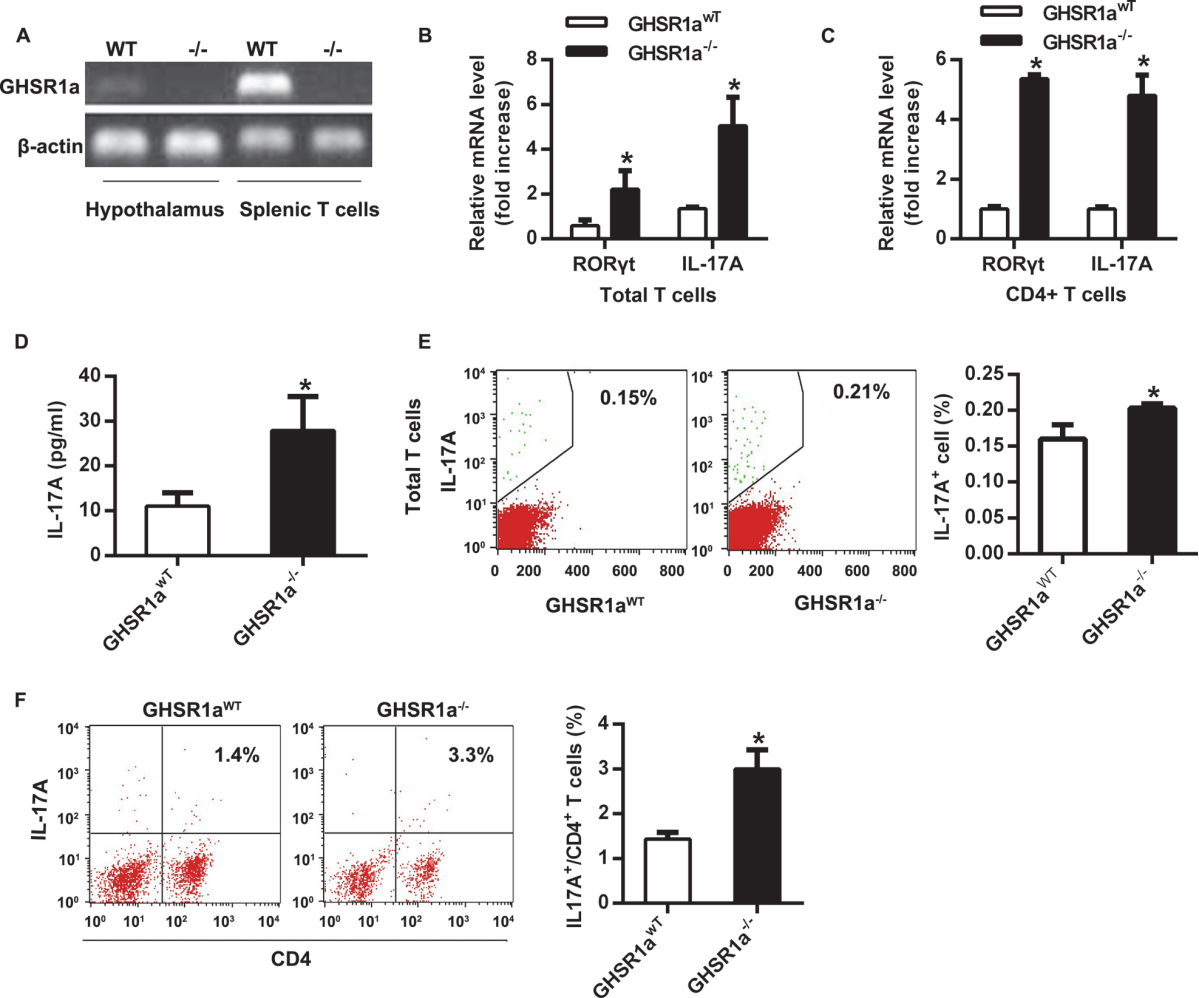
Enhanced differentiation of Th17 cells in GHSR1a^-/-^ mice. 8 to 10-wk-old GHSR1a^WT^ (n = 5) and GHSR1a^-/-^ (n = 6) male mice were fed standard chow. (A) Total T cells were isolated from the spleen of GHSR1a^WT^ and GHSR1a^-/-^ mice. The mRNA level of GHSR1a was evaluated to verify the deficiency of GHSR1a. (B&C) The mRNA levels of RORγt and IL-17A in splenic total T cells and CD4^+^ T cells were analyzed with RT-PCR. (D) The concentration of IL-17A in serum was examined with ELISA. (E&F) The percentage of IL-17A^+^ cells in splenic total T cells (E) and CD4^+^ T cells (F) was analyzed with flow cytometry. Shown is the representative of three independent experiments. *P<0.05 versus wild type control.

To confirm the loss of GHSR1a, splenic T cells from GHSR1a^-/-^ mice and wide type littermates were analyzed for GHSR expression by PCR [24]. GHSR1a was detected only in spenic T cells derived from wildtype mice (Fig. 1A). RORγt is a critical transcription factor for Th17 cell development and IL-17A is a hallmark cytokine secreted from Th17 cells [2,4,18]. Compared with GHSR1a^WT^ mice, the mRNA expression of RORγt and IL-17A was greatly increased in spleens of GHSR1a^-/-^ mice both in splenic total T cells and CD4^+^ T cells (Fig. 1B & C). However, the expression of other lineage factors, such as FoxP3, GATA3, Tbx21 and IFNγ was not affected (S2 Fig.). Serum level of IL-17A also increased in GHSR1a^-/-^ mice (Fig. 1D). We also analyzed the splenic content of Th17 cells. Splenic cell sorting by flow cytometry also showed a significant increase in IL17A^+^ cells in GHSR1a^-/-^ mice both in splenic total T cells and CD4^+^ T cells (Fig. 1E & F). These results suggested that the differentiation of Th17 cells increased in spleen of ghrelin receptor GHSR1a knockout mice.

### Ghrelin inhibited the differentiation of Th17 cells *in vitro*

**Fig 2:**
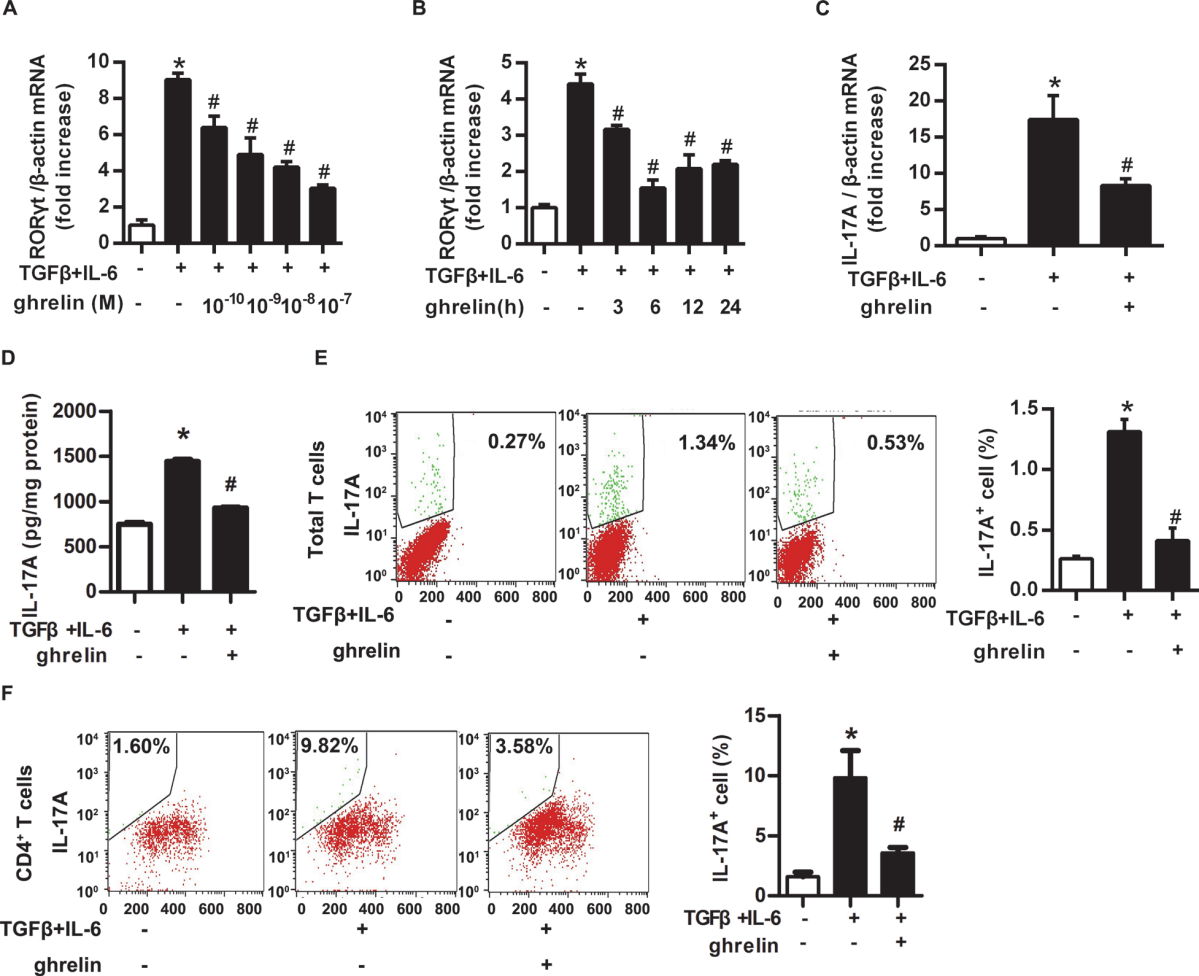
Ghrelin inhibited the differentiation of Th17 cells *in vitro*. (A&B) Total T cells were isolated from mouse spleens and induced to differentiate into Th17 cells with TGF-β (5 ng/ml) and IL-6 (20 ng/ml), then treated with ghrelin with final concentration (A) and time (B) as indicated. The mRNA level of RORγt was analyzed with RT-PCR. (C) Differentiated Th17 cells were treated with ghrelin (10^–8^ M). The mRNA level of IL-17A was analyzed with RT-PCR. (D)The concentration of IL-17A in the supernatant was examined with ELISA. (E&F) Cells were stimulated with PMA, Ionomycin and Brefeldin A for 4–6 hours. The percentage of IL-17A^+^ cells in splenic total T cells (E) and CD4^+^ T cells (F) was analyzed with flow cytometry. Shown is the representative of three independent experiments. *P<0.05 versus control; ^#^ *P*<0.05 versus ghrelin-treated alone.

We then tested the direct effect of ghrelin on the differentiation of Th17 cells. Ghrelin (10^-10^-10^-7^M) reduced the mRNA level of RORγt in a concentration- and time- dependent manner, with maximal inhibition at 6h (Fig. 2A & B). The mRNA and protein levels of IL-17A also significantly reduced after ghrelin treatment (Fig. 2C & D). Similar results were also observed in ghrelin-treated C57BL/6 mice, without being affected by physiological fluctuation of ghrelin (S3 & S4 Figs.). Consistent with the changes of RORγt and IL-17A, cell sorting by flow cytometry suggested that IL-17A^+^ T cell number was significantly reduced under ghrelin treatment both in splenic total T cells and CD4^+^ T cells (Fig. 2E&F). These data demonstrated that ghrelin could inhibit the differentiation of Th17 cells *in vitro*.

### mTOR signaling pathway mediated the inhibitory effect of ghrelin on Th17 cells

**Fig 3:**
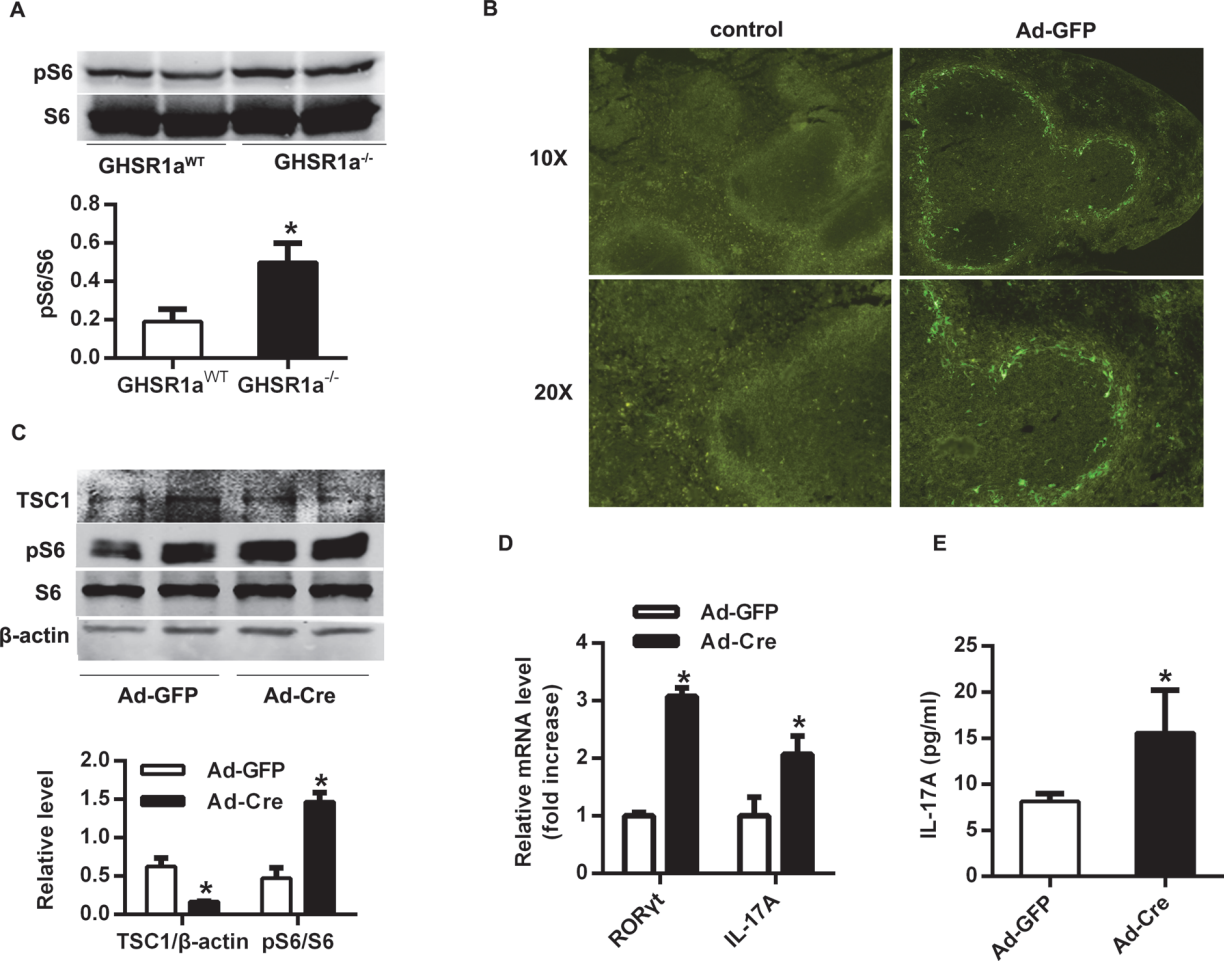
mTOR signaling pathway mediated the inhibitory effect of ghrelin on Th17 cells. (A) Total T cells were isolated from spleens of GHSR1a ^WT^ and GHSR1a^-/-^ mice. The phosphorylation of S6 was analyzed with Western Blot. Relative protein signal intensity was quantified. (B) 8 to 10-wk-old TSC1^loxp/loxp^ mice were injected intravenously through caudal vein with GFP or Cre virus for 4 weeks. The location of Ad-GFP in spleen was observed using immunoluorescence microscopy. (C) Total T cells were isolated from the spleens of mice injected with Ad-GFP or Ad-Cre. The expression of TSC1 and the phosphorylation of S6 were analyzed with Western Blot. Relative protein signal intensity was quantified. (D) Total T cells were isolated from the spleens of mice injected with Ad-GFP and Ad-Cre. The mRNA levels of RORγt and IL-17A were analyzed with RT-PCR. (E) The concentration of IL-17A in mice serum was examined with ELISA. *P<0.05 versus Ad-GFP injected group.

mTOR had been shown to integrate immune microenvironment with the differentiation of T cells [20]. Ghrelin, which plays an important role in energy homeostasis, has been reported to regulate mTOR signaling [25,26,27]. We therefore analyzed the phosphorylation of S6, the downstream molecule of mTOR, in GHSR1a^-/-^ T cells. As shown in Fig. 3A, phosphorylation of S6 was up-regulated in GHSR1a^-/-^ T cells, indicating that ghrelin might inhibit the differentiation of Th17 cells through mTOR signaling pathway. We then injected Ad-Cre into TSC1^loxp/loxp^ mice through the tail vein to knock down the expression of TSC1, an important upstream inhibitor of mTOR. Through fluorescence microscope, we observed green florescence in spleen which suggested that adenovirus could distribute in spleen (Fig. 3B). Compared with the group injected with Ad-GFP, the expression of TSC1 reduced and phosphorylated S6 increased significantly in T cells from the mice injected with Ad-Cre (Fig. 3C). This observation confirmed that the activation of mTOR signaling by knockdown the TSC1 in splenic T cells of Ad-Cre injected group. The alteration in mTOR signaling was accompanied by a significantly increase in mRNA levels of RORγt and IL-17A (Fig. 3D), and serum levels of IL-17A (Fig. 3E). These results suggested that mTOR signaling pathway might mediate the inhibitory effect of ghrelin on Th17 cells.

### Leucine rescued the inhibitory effect of ghrelin on Th17 cells

**Fig 4:**
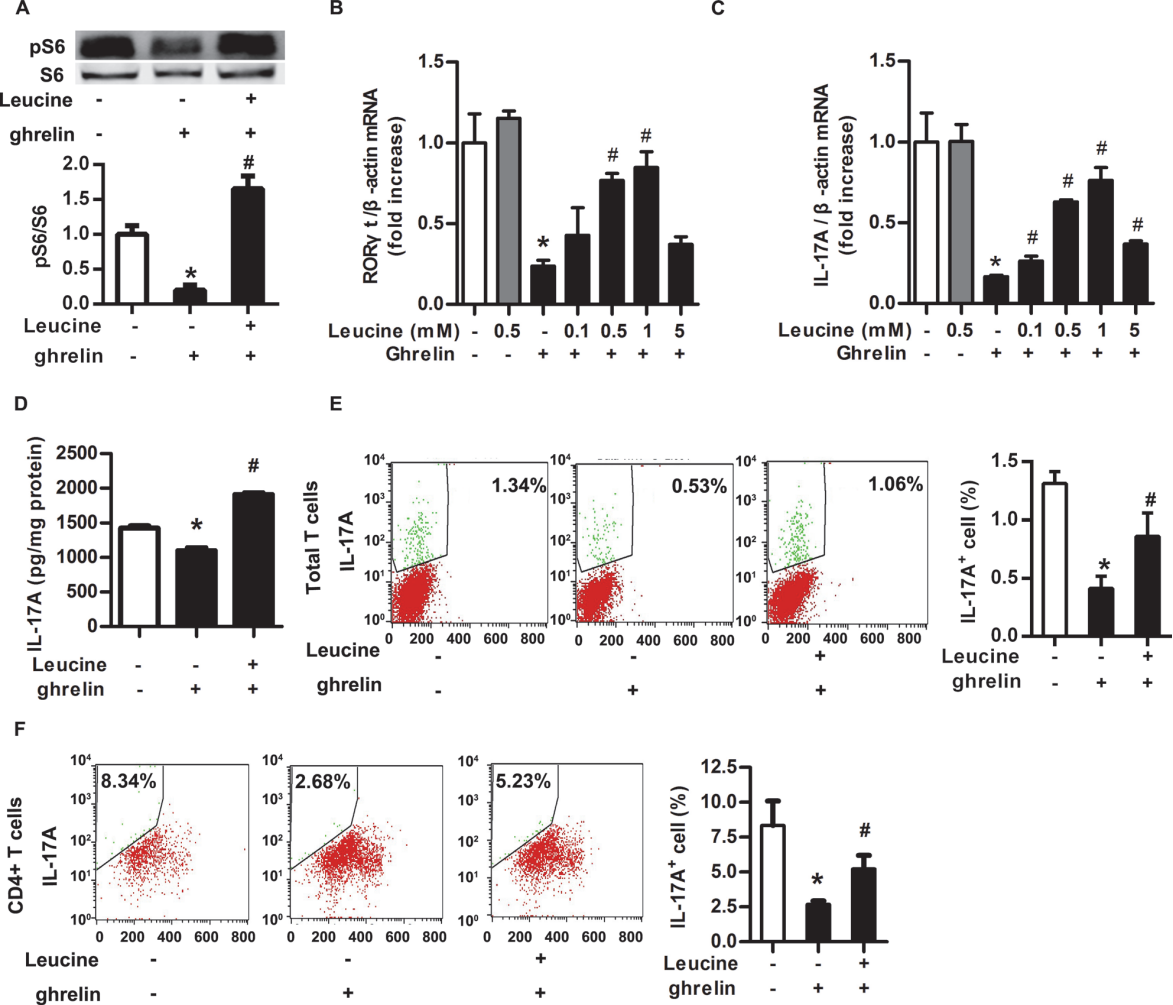
Leucine rescued the inhibitory effect of ghrelin on Th17 cells. Total T cells were isolated from mouse spleens and induced differentiation to Th17 cells. (A) Differentiated Th17 cells were treated with ghrelin (10^–8^ M) and leucine (1 mM). The phosphorylation of S6 was analyzed with Western Blot. Relative protein signal intensity was quantified. (B&C) Differentiated Th17 cells were treated with ghrelin (10^–8^ M) and leucine with final concentration as indicated. The mRNA levels of RORγt (B) and IL-17A (C) were analyzed with RT-PCR. (D) Differentiated Th17 cells were treated with ghrelin (10^–8^ M) and leucine (1 mM). The concentration of IL-17A in the supernatant was examined with ELISA. (E&F) The percentage of IL-17A^+^ cells in splenic total T cells (E) and CD4^+^ T cells (F) was analyzed with flow cytometry. Shown is the representative of three independent experiments. *P<0.05 versus control; ^#^ *P*<0.05 versus ghrelin-treated alone.

To confirm whether the activation of mTOR mediates the inhibitory effect of ghrelin on Th17 cells, leucine, the activator of mTOR signaling pathway, was used. T cells from spleen of wide type mice were induced to differentiate in the presence of TGF-β1 and IL-6, preincubated with or without leucine for 3 hours before treatment with ghrelin. Leucine neutralized the inhibitory effect of ghrelin on mTOR signaling pathway (Fig. 4A). Increasing concentrations of leucine progressively reversed the expression of RORγt and IL-17A inhibited by ghrelin (Fig. 4B & C). As well, ELISA and FACS analysis indicated that the reduced concentration of IL-17A in supernatant and IL-17A^+^ T cells were rescued by the addition of leucine both in splenic total T cells and CD4^+^ T cells (Fig. 4D-F). Thus, activation of mTOR signaling pathway by leucine could rescue the inhibitory effect of ghrelin on the differentiation of Th17 cells.

### Overexpression of S6K1 rescued the inhibitory effect of ghrelin on Th17 cells

**Fig 5:**
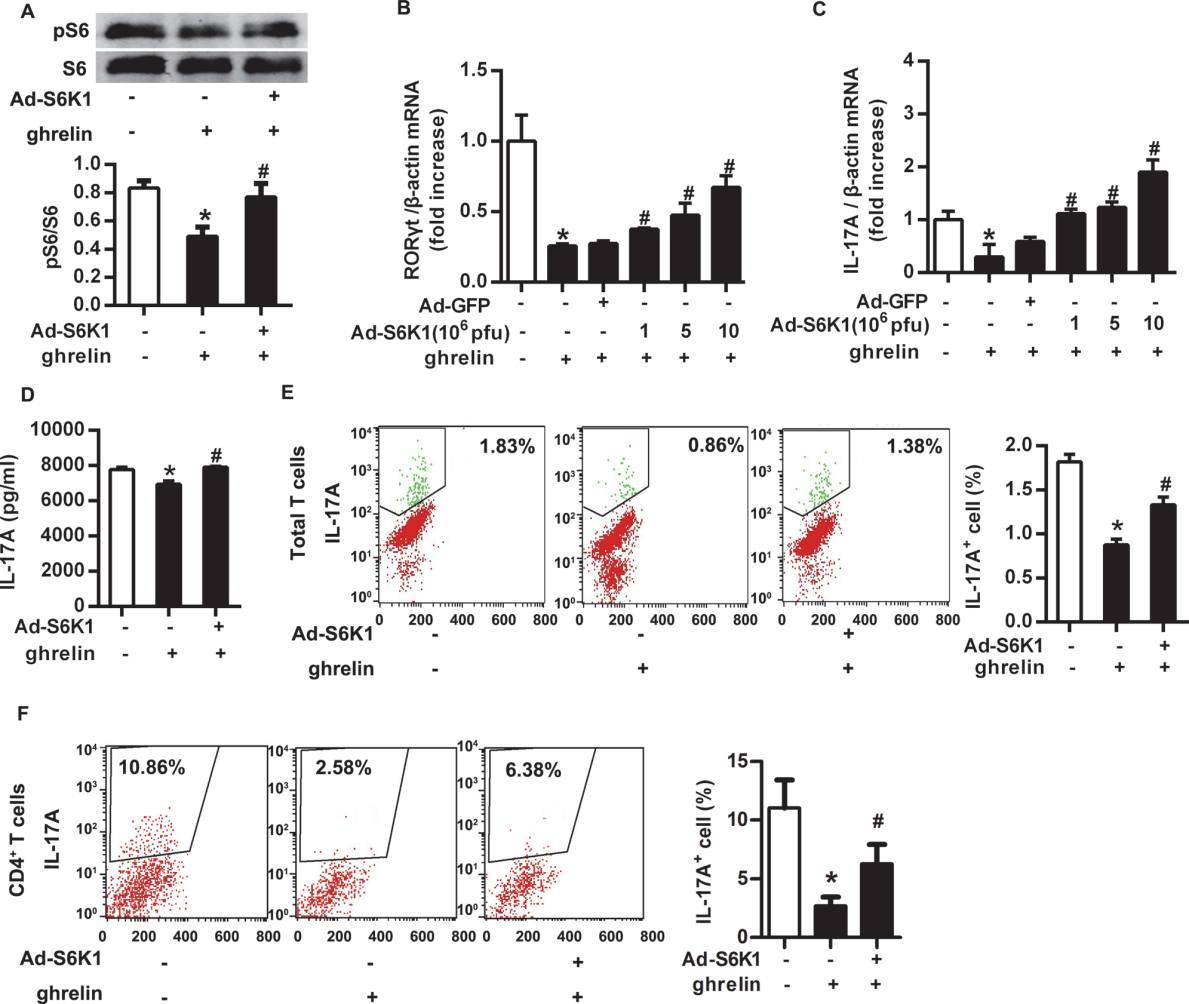
Overexpression of S6K1 rescued the inhibitory effect of ghrelin on Th17 cells. Total T cells were isolated from mouse spleens and induced differentiation to Th17 cells. Adenovirus was used to infect activated T cells to overexpress S6K1, the critical downstream molecule in mTOR signaling pathway. (A) The phosphorylation of S6 was analyzed with Western Blot. Relative protein signal intensity was quantified. (B&C) Differentiated Th17 cells were treated with adenovirus expressing S6K1 (Ad-S6K1) or GFP (Ad-GFP) with final titer indicated. The mRNA level of RORγt (B) and IL-17A (C) were analyzed with RT-PCR. (D) The concentration of IL-17A in the supernatant was examined with ELISA. (E&F) The percentage of IL-17A^+^ cells in splenic total T cells (E) and CD4^+^ T cells (F) was analyzed with flow cytometry. Shown is the representative of three independent experiments. *P<0.05 versus control; ^#^ *P*<0.05 versus ghrelin-treated alone.

S6K1 is an important downstream molecule of mTOR, which phosphorylates S6. Western Blot confirmed that Ad-S6K1 infection reversed the inhibited phosphorylation of S6 caused by ghrelin (Fig. 5A). Similar with the supplement of leucine, Ad-S6K1 infection could titer-dependently reverse the down-regulated mRNA expression of RORγt and IL-17A by ghrelin, and increased the secretion of IL-17A in supernatant (Fig. 5B-D). Cell sorting by flow cytometry suggested that ghrelin-treated IL-17A^+^ T cell number was significantly restored by Ad-S6K1 infection both in splenic total T cells and CD4^+^ T cells (Fig. 5E & F). Thus, activation of mTOR signaling pathway rescued the inhibitory effect of ghrelin on Th17 cells.

### STAT3 signaling pathway mediated the inhibitory effect of ghrelin on Th17 cells

**Fig 6:**
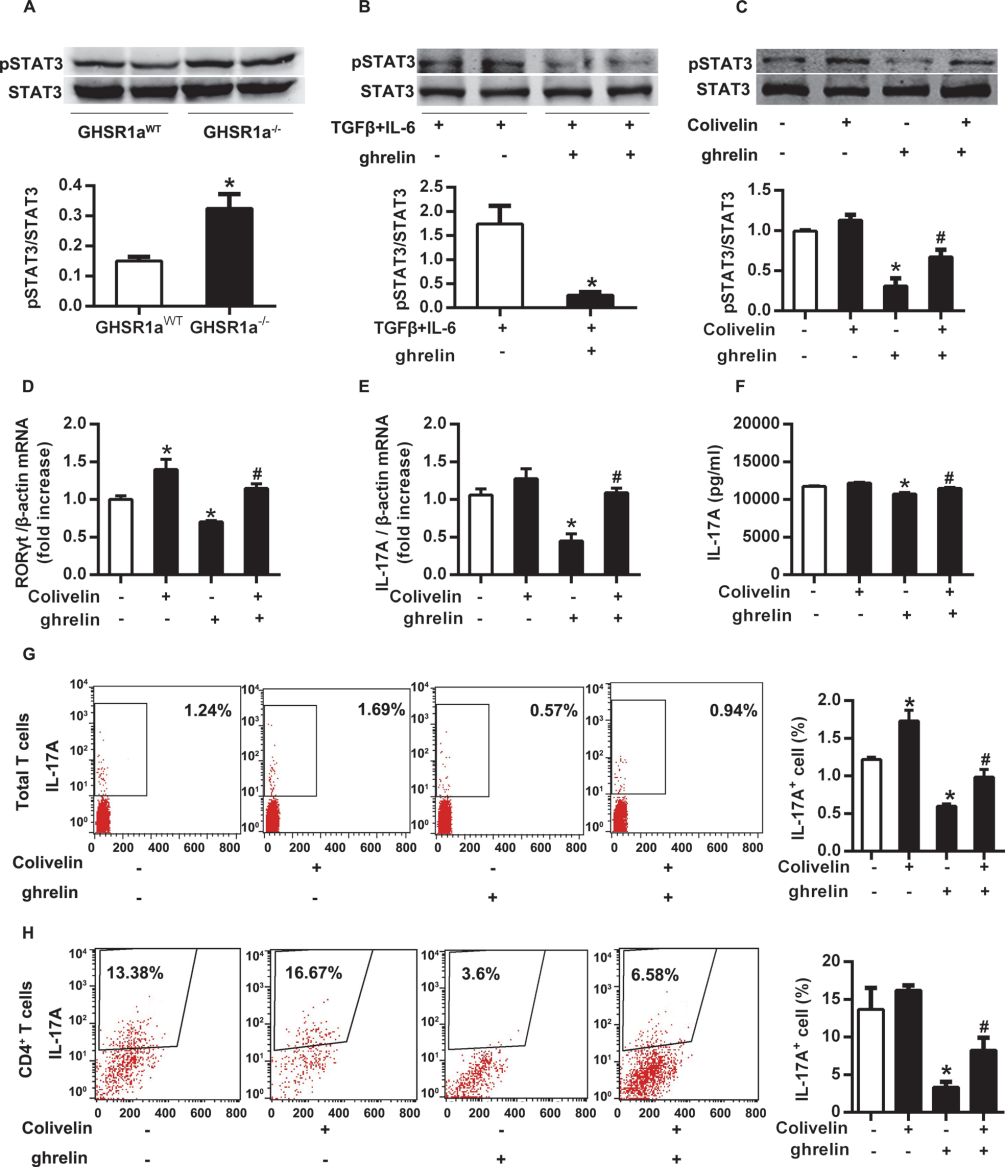
STAT3 signaling pathway was involved in the inhibitory effect of ghrelin on Th17 cells. (A) Total T cells were isolated from spleens of GHSR1a^WT^ and GHSR1a^-/-^ mice. The phosphorylation of STAT3 was analyzed with Western Blot. (B) Total T cells were isolated from mouse spleens and induced differentiation to Th17 cells, then treated with ghrelin (10^–8^ M). The phosphorylation of STAT3 was analyzed with Western Blot. (C) Differentiated Th17 cells were pre-treated with or without Colivelin (100 pM), then treated with or without ghrelin (10^-8^M). The phosphorylation of STAT3 was analyzed with Western Blot. Relative protein signal intensity was quantified. (D&E) The mRNA level of RORγt (D) and IL-17A (E) was analyzed with RT-PCR. (F) The concentration of IL-17A in the supernatant was examined with ELISA. (G&H) The percentage of IL-17A^+^ cells in splenic total T cells (G) and CD4^+^ T cells (H) was analyzed with flow cytometry. Shown is the representative of three independent experiments. *P<0.05 versus control; ^#^ *P*<0.05 versus ghrelin-treated alone.

STAT3 has emerged as an important regulator of the differentiation of Th17 cells [28]. Next, we determined whether ghrelin influences the activation of STAT3. In GHSR1a^-/-^ T cells, we observed increased activation of STAT3 (Fig. 6A). The level of phosphorylated STAT3 also reduced upon ghrelin stimulation *in vitro* (Fig. 6B). These data indicated that ghrelin might exert its inhibitory effect on Th17 cells through reducing the activation of STAT3. To rest this concept, we pre-treated expanded Th17 cells with or without Colivelin before stimulation with ghrelin. Treatment with Colivelin for 16 hours activated STAT3 (Fig. 6C). Colivelin upregulated the inhibited expression of RORγt and IL-17A by ghrelin in both mRNA and protein level (Fig. 6D-F). FACS analysis of IL-17A^+^ T cells also indicated that Colivelin increased the number of IL17A^+^ T cells and rescued the inhibitory effect of ghrelin on Th17 cells both in total splenic T cells and CD4^+^ T cells (Fig. 6G & H). Therefore, STAT3 signaling pathway might mediate inhibitory effect of ghrelin on Th17 cells.

### STAT3 was positively regulated by mTOR

**Fig 7:**
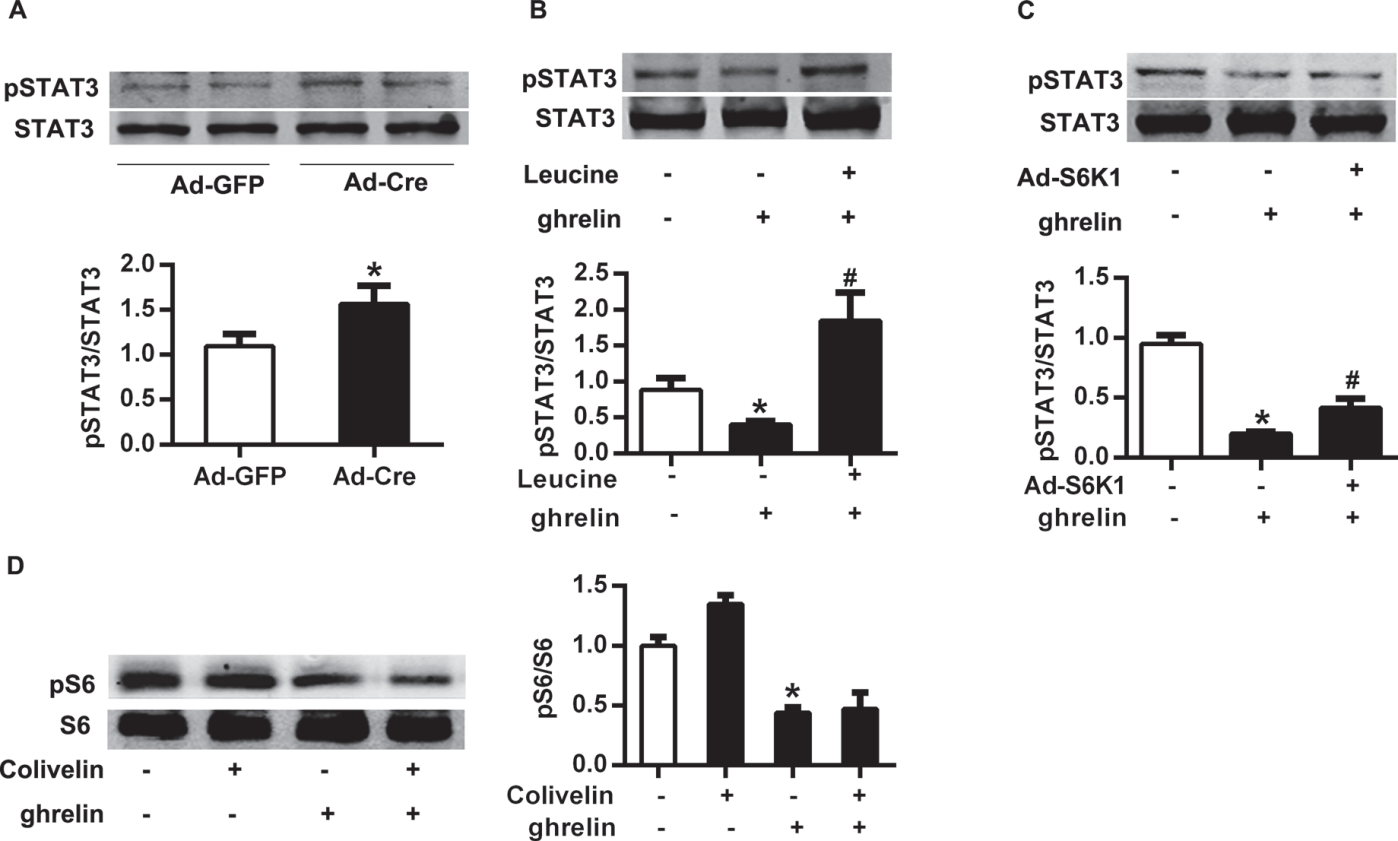
STAT3 was positively regulated by mTOR. (A) Total T cells were isolated from the spleen of TSC1^loxp/loxp^ mice injected with Ad-GFP and Ad-Cre. The phosphorylation of STAT3 was analyzed with Western Blot. (B) Differentiated Th17 cell was stimulated with ghrelin and/or leucine. The phosphorylation of STAT3 was analyzed with Western Blot. (C) Adenovirus was used to infect differentiated Th17 cells to overexpress S6K1. The phosphorylation of STAT3 was analyzed with Western Blot. (D) Differentiated Th17 cells were stimulated with ghrelin and/or colivelin. The phosphorylation of S6 was analyzed with Western Blot. Relative protein signal intensity was quantified.

Since both mTOR and STAT3 signaling were indicated to be important for ghrelin-inhibited differentiation of Th17 cells, we next explored the interaction between the two pathways. Compared with TSC1^loxp/loxp^ mice injected with Ad-GFP, the phosphorylation level of STAT3 increased in splenic T cells of the mice injected with Ad-Cre (Fig. 7A). Thus, mTOR might positively regulate the activation of STAT3. In addition, supplement of leucine or infection of Ad-S6K1 could rescue the reduction of phosphorylated STAT3 caused by stimulation of ghrelin in T cells (Fig. 7B & C). However, the phosphorylation of S6 was not altered in Th17 cells stimulated with STAT3 activitor Colivelin (Fig. 7D). These experiments suggested that STAT3 is positively regulated by mTOR, and mTOR-STAT3 signaling pathway might mediate the inhibitory effect of ghrelin on Th17 cells.

## Discussion

The present study demonstrates that ghrelin can inhibit the differentiation of Th17 cells via mTOR/STAT3 signaling pathway. This conclusion is supported by the following observations: (1) mRNA expression of RORγt, IL-17A and numbers of IL-17A^+^ T cells are greatly increased in spleens of GHSR1a^-/-^ mice; (2) ghrelin reduces the mRNA and protein levels of RORγt and IL-17A *in vitro* in a concentration dependent manner, while IL-17A^+^ T cell number is significantly reduced under ghrelin treatment; (3) mTOR and STAT3 activation is inhibited in GHSR^-/-^ mice and by ghrelin treatment *in vitro*; (4) activation of mTOR or STAT3 signaling pathway reverses the inhibitory effect of ghrelin on Th17 cells; (5) STAT3 is positively regulated by mTOR activation. To the best of our knowledge, this is the first report demonstrating the involvement of multiple signaling pathways in the ghrelin-mediated modulation of Th17 cell differentiation.

Th17 cells play an important role in autoimmune and inflammatory diseases. Ghrelin can suppress the expression of pro-inflammation cytokines to exert its anti-inflammatory function. Recent study suggests that activated T cells which have reduced expression of ghrelin have enhanced expression of pro-inflammation cytokines including IL-17. In mice, ghrelin treatment prevents EAE-induced infiltration of Th17 and Th1, and alleviates brain damage [17,29]. Our research further confirms the inhibitory effect of ghrelin on Th17 cells.

mTOR is emerging as critical regulator of immune function because its role in sensing and integrating cues from the immune microenvironment. T cells lacking mTOR fail to differentiate into Th17 cells under polarizing condition. This failure to differentiate is associated with decreased expression of RORγt. In our experiments, addition of ghrelin inhibits the differentiation of Th17 cells and down-regulates the phosphorylation of S6, the downstream molecule of mTOR. Activation of mTOR signaling pathway by either leucine stimulation, Ad-S6K1 infection or TSC1 knockdown all reverses the inhibition effect of ghrelin on Th17 cells. Therefore, ghrelin regulates the differentiation of Th17 cells through mTOR signaling pathway. Previous observations suggest that mTOR mediates the effects of ghrelin on lipid synthesis in hepatic cells, food intake, and intestinal ischemia-reperfusion [25,26,27]. Yet we found that ghrelin reduces the activation of mTOR signaling pathway in activated T cells. Ghrelin might suppress mTOR activation through an yet-to-be-identified mechanisms.

Phosphorylation of STAT3, the downstream signal of IL-6 and IL-21, is a key process in the differentiation of Th17 cells in the presence of TGF-β [3]. In the Th17-inducing conditions, ghrelin inhibits the differentiation of Th17 cells and the phosphorylation of STAT3. Therefore, ghrelin may reduce the differentiation of Th17 cells through inhibiting STAT3 phosphorylation. It was reported that the expression of STAT3 decreases in breast cancer cells transfected with lentivirus-based shRNAs targeting the mTOR gene [30]. We also found that downregulation of TSC1, the natural inhibitor of mTOR, is associated with the increased activation of STAT3. However, the phosphorylation of S6 did not altered after the activation of STAT3 and the reduced activation of S6 caused by ghrelin was not reversed by using STAT3 activator. These data suggest that STAT3 is the downstream of mTOR in the Th17 cells.

The promise of discovering strategy modulating the differentiation of Th17 cells lies in the potential to provide new therapeutic approaches for autoimmune and inflammatory diseases. We have found that ghrelin suppresses the differentiation of Th17 cells *in vivo* and *in vitro*. In particular, we demonstrate that mTOR/STAT3 signaling might mediate the inhibitory effect of ghrelin on the differentiation of Th17 cells. Thus, ghrelin, an important gastrointestinal hormone that regulates metabolism, is now recognized as an immune factor that regulates immune homeostasis.

## Supporting Information

S1 Fig Splenic CD4^+^ T cells were isolated from the spleens of C57BL/6J mice and induced to differentiate into Th17 cells with TGF-β (5 ng/ml) and IL-6 (20 ng/ml). The percentages of IL-17A^+^ cells and FoxP3^+^ cells were analyzed with flow cytometry. Shown is the representative of three independent experiments.
(TIF)

S2 Fig 8 to 10-wk-old GHSR1a^WT^ (n = 5) and GHSR1a^-/-^ (n = 6) male mice were fed standard chow. Total T cells were isolated from the spleen of GHSR1a^WT^ and GHSR1a^-/-^ mice. The mRNA levels of FoxP3, GATA3, Tbx21 and IFNγ were analyzed with RT-PCR, normalized to internal control β-actin and expressed as mean±SEM.
(TIF)

S3 Fig Total T cells were isolated from the spleens of C57BL/6J mice with or without fasting for 24 hours. The mRNA levels of RORγt and IL-17A were analyzed with RT-PCR, normalized to internal control β-actin and expressed as mean±SEM.
(TIF)

S4 Fig C57BL/6J were were injected intraperitoneally with LPS (80 μg/Kg/day) for 7 days, with or without ghrelin administration by smotic pumps. Total T cells were isolated from the spleens of mice. The mRNA levels of RORγt and IL-17A were analyzed with RT-PCR, normalized to internal control β-actin and expressed as mean±SEM. *P<0.05 versus control; ^#^ *P*<0.05 versus LPS-treated alone.
(TIF)
