# Supplementary figures and images for: Ghrelin Inhibits the Differentiation of T Helper 17 Cells through mTOR/STAT3 Signaling Pathway
Source: PLoS One. 2015 Feb 6;10(2):e0117081. doi: 10.1371/journal.pone.0117081 (PMC4319964; doi:10.1371/journal.pone.0117081)

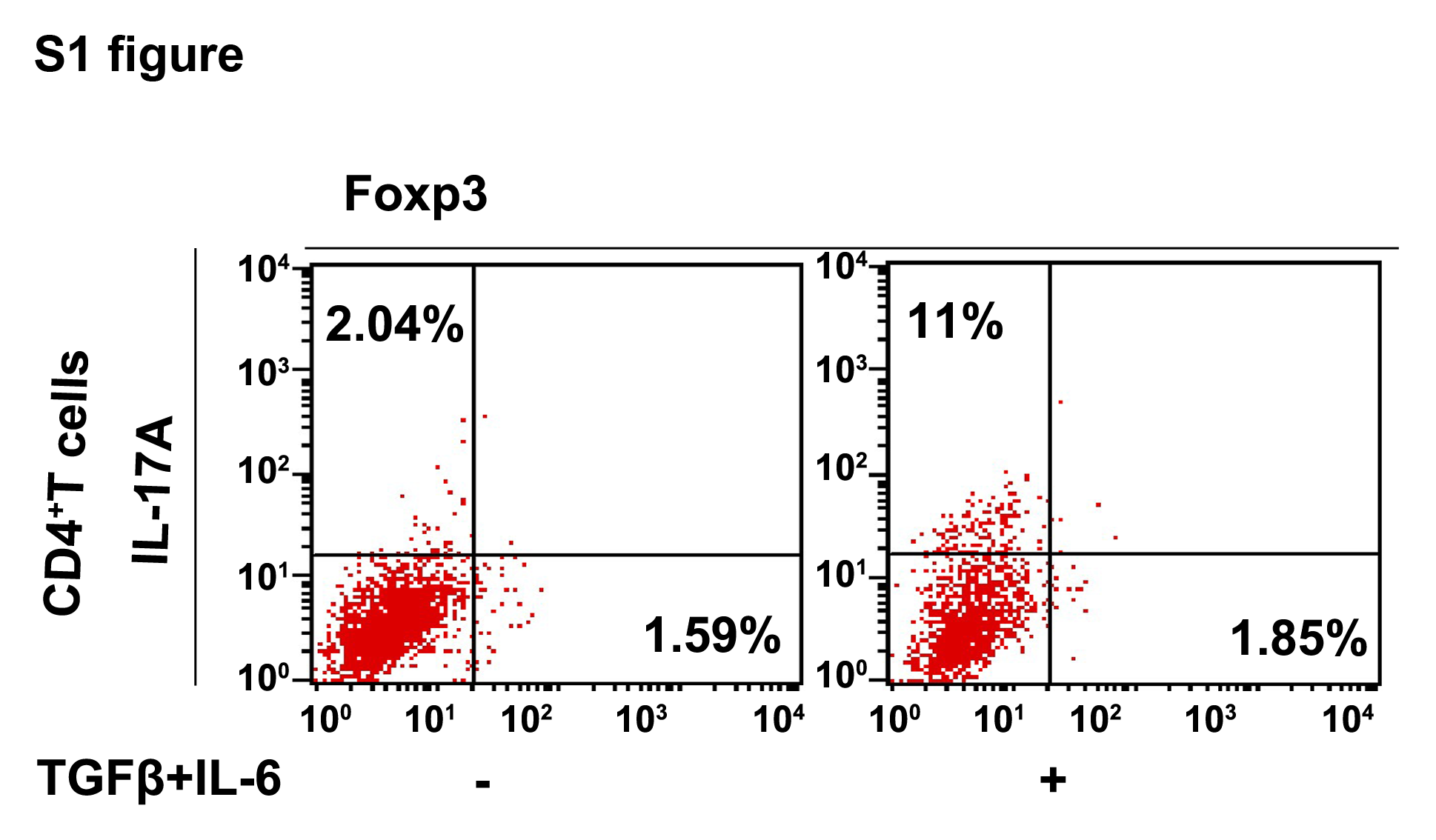

Supplement: S1 Fig — The percentages of IL-17A+ cells and FoxP3+ cells were analyzed with flow cytometry. Shown is the representative of three independent experiments. (TIF) [file pone.0117081.s001.tif]

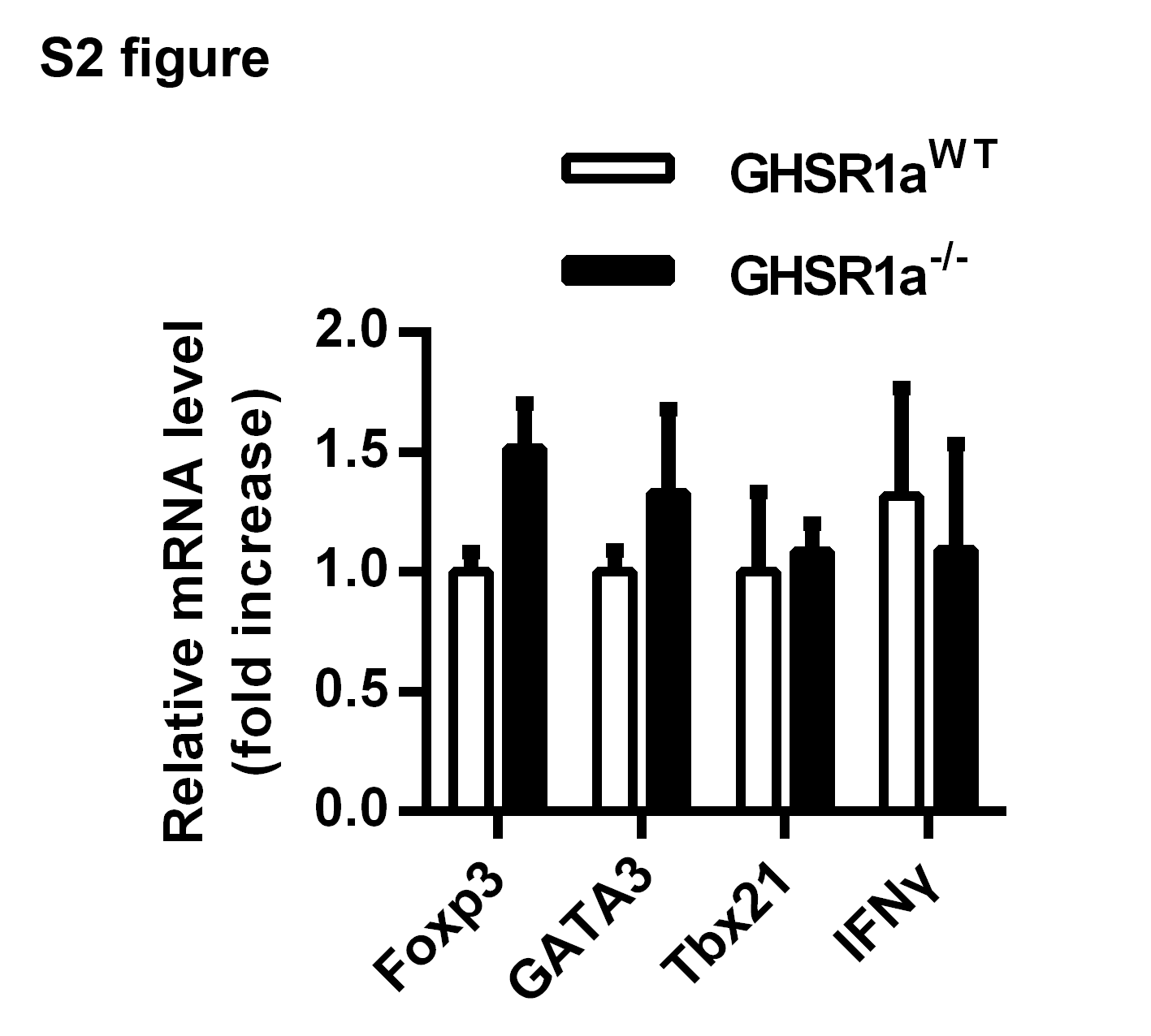

Supplement: S2 Fig — Total T cells were isolated from the spleen of GHSR1aWT and GHSR1a-/- mice. The mRNA levels of FoxP3, GATA3, Tbx21 and IFNγ were analyzed with RT-PCR, normalized to internal control β-actin and expressed as mean±SEM. (TIF) [file pone.0117081.s002.tif]

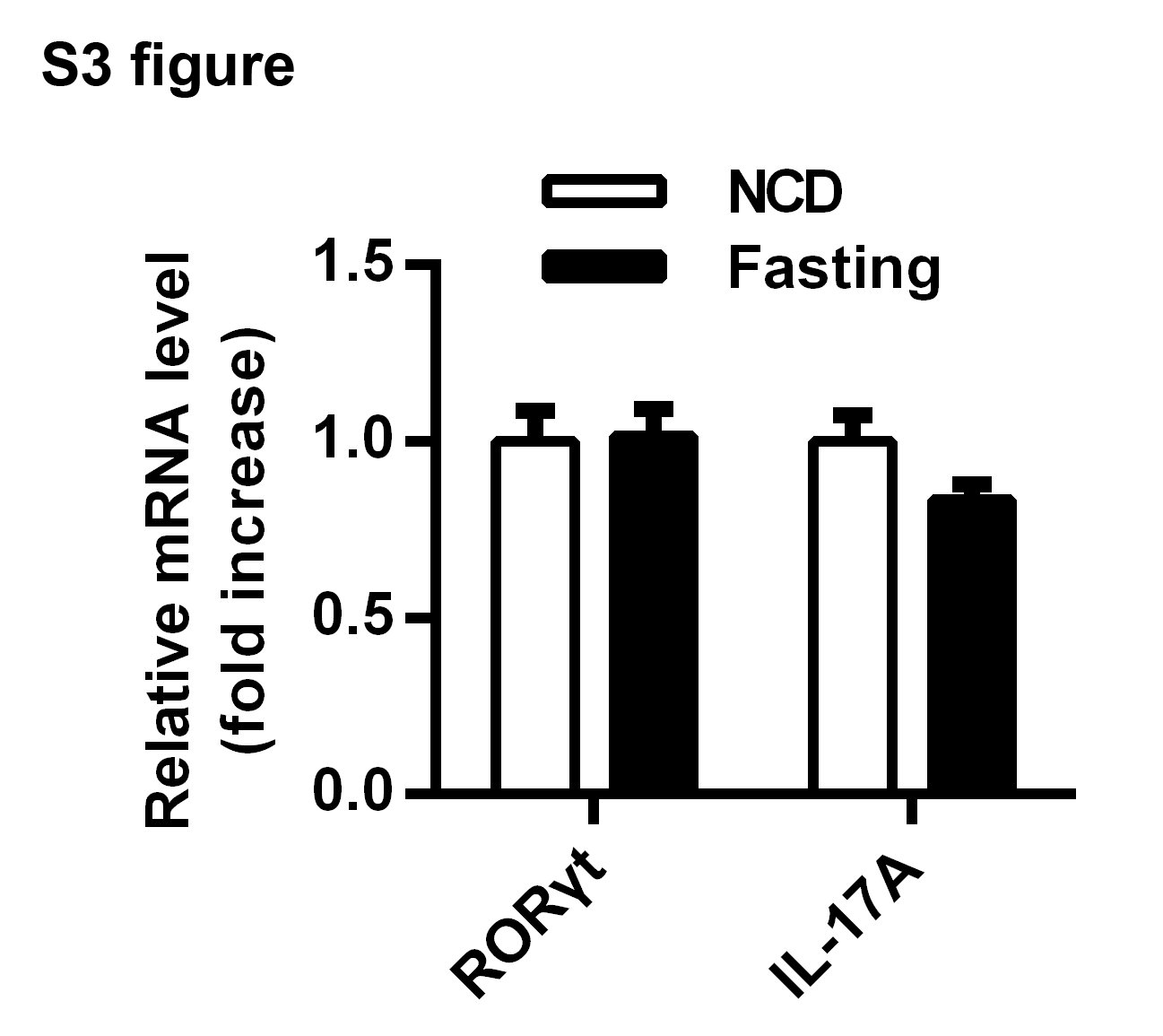

Supplement: S3 Fig — The mRNA levels of RORγt and IL-17A were analyzed with RT-PCR, normalized to internal control β-actin and expressed as mean±SEM. (TIF) [file pone.0117081.s003.tif]

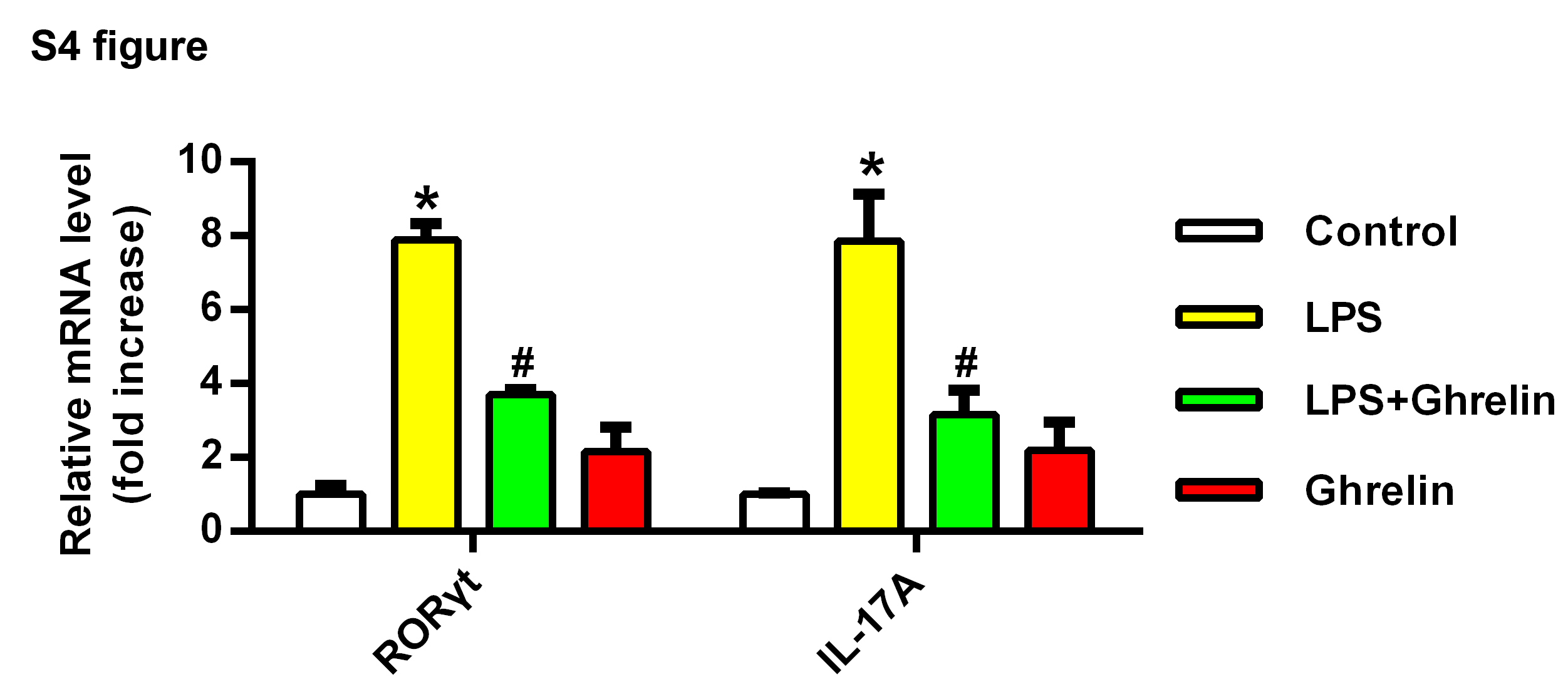

Supplement: S4 Fig — Total T cells were isolated from the spleens of mice. The mRNA levels of RORγt and IL-17A were analyzed with RT-PCR, normalized to internal control β-actin and expressed as mean±SEM. *P<0.05 versus control; # P<0.05 versus LPS-treated alone. (TIF) [file pone.0117081.s004.tif]
